# A retrospective analysis of pegylated liposomal doxorubicin in ovarian cancer: do we still need it?

**DOI:** 10.1186/1757-2215-6-10

**Published:** 2013-02-06

**Authors:** Nicoletta Staropoli, Domenico Ciliberto, Cirino Botta, Lucia Fiorillo, Simona Gualtieri, Angela Salvino, Pierfrancesco Tassone, Pierosandro Tagliaferri

**Affiliations:** 1 Medical Oncology Unit, Department of Experimental and Clinical Medicine, Magna Græcia University and Tommaso Campanella Cancer Center, Campus Salvatore Venuta, Catanzaro, Italy

**Keywords:** Ovarian cancer, Systemic chemotherapy, Pegylated liposomal doxorubicin, Second line treatment, Platinum refractory patients

## Abstract

**Background:**

Ovarian cancer (OC) is the sixth most common cancer in women. Currently, carboplatin/paclitaxel ± bevacizumab is the cornerstone of front-line treatment. Conversely, the therapeutic options for recurrent or progressive disease are not well defined. For platinum-sensitive patients the best therapeutic approach is still a re-challenge with a platinum-based regimen. Pegylated liposomal doxorubicin (PLD), is considered one of the most active therapeutic options for recurrent or progressive OC. In this retrospective mono-institutional analysis, we evaluated the impact of PLD on the outcome of OC patients.

**Patients and methods:**

We performed the retrospective study on a cohort of 108 patients with histologically confirmed serous papillary OC, followed at our Institution between 2001 and 2011. 80 patients were in stage III/IV and 55 of them received a second-line treatment. Thirty patients were treated with PLD. Both groups (PLD-treated *versus* PLD-untreated) underwent a median of 3 treatment lines and were prognostically balanced. The median follow-up was 60 months. Survival endpoints, toxicity and correlations between patients’ baseline characteristics and treatment efficacy were evaluated.

**Results:**

Patients who had undergone PLD treatment (PLD group) showed a median overall survival (OS) of 45 months as compared to 65 months of patients not treated with PLD (PLD-free group) (HR 2.50 [0.95-6.67; p = 0.06]). Moreover, the median progression-free survival was 6 months in the PLD group *versus* 10 months in the PLD-free group (HR 1.75 [0.94-3.34; p = 0.07]). The overall objective response rate in II line treatment was 43% (13% in PLD group *versus* 57% in PLD-free group). Furthermore, we investigated survival endpoints in platinum-refractory patients who received PLD at least once during the course of disease. No OS advantage was achieved by PLD administration when compared to other therapeutic options (30 *versus* 32 months; HR 1.16 [0.31-4.34; p = 0.81]). No difference in term of toxicity was observed among different groups.

**Conclusions:**

No evidence of superiority if PLD was compared to alternative agents was found in this analysis, particularly in the platinum-refractory setting. Our findings indicate a modest therapeutic activity of PLD in OC. Analysis of cost/benefit of PLD in OC is eagerly awaited.

## Background

Ovarian cancer (OC) is the sixth most common malignancy in women [1]. Serous papillary OC, the most frequent histotype, is the leading cause of death for gynecological tumors and represents 5% of cancer-related mortality in women in the western world. The overall 5-years survival rate is 30% [2] and about 2/3 of OC patients are diag-nosed in an advanced stage (stage II with residual disease, stage III and stage IV). FIGO (International Federation of Gynecology and Obstetrics) stage, surgical residual disease and histological grade, at diagnosis, are known to influence the outcome of these patients [3,4].

In the last years, a variety of clinical trials failed to demonstrate advantages of the addition of a third cytotoxic drug to the standard doublet [5,6] and currently, carboplatin/paclitaxel ± bevacizumab is the cornerstone of front-line treatment [7,8]. Conversely, the therapeutic scenario for recurrent or progressive disease remains still undefined. Second-line therapy is selected taking into account the response to front-line platinum-based treatment. Patients who relapse within 6 months from the last platinum-based treatment course are considered platinum-refractory, while patients who relapse at least 12 months after the last chemotherapy course are considered platinum-sensitive. Furthermore, it is common practice to consider a third group of patients, the “partially platinum-sensitive patients”, as those who relapse between 6 and 12 months [9,10]. This clinical classification appears to better represent the clinical outcome of serous papillary OC as compared to mucinous or clear cell or low grade endometrioid OC [11], probably due to a different embryologic origin and pathogenesis. It has been hypothesized that serous papillary OC directly arises from the ovary and tube epithelium while endometrioid or mucinous may derive from endometrial or gastrointestinal embrional implants, respectively [12,13]. Moreover, it has been described a strong association between platinum-sensitivity and high grade serous papillary OC, harboring p53 mutation and somatic or germline mutations of BRCA1 [13]. This observation founds its molecular basis in the failure of the DNA repair homologous recombination mechanisms (due to loss of BRCA1 function) to restore double-strand breaks induced by platinum compounds, and has been described in experimental *in vitro* and *in vivo* models [14-18].

Concerning the second-line therapy for platinum-sensitive patients, a re-challenge with a carboplatin-based regimen is considered the optimal approach [19]. Two randomized trials (GOG 182 and Calypso trials), that included all OC histotypes, demonstrated an advantage for carboplatin/PLD combination as compared to carboplatin/paclitaxel in both front-line and second-line therapy in terms of progression free survival (PFS) [6,20-23]. PLD became therefore a primary option in clinical practice, taking also in account the good safety profile.

On the other hand, no standard approach for second line treatment exists for platinum-refractory patients [24,25]. In the past years, different phase II/III trials investigated the activity of several drugs such as PLD, topotecan, etoposide, taxanes, gemcitabine, oxaliplatin in this group of patients; however clinical results were modest with low response rates (RR) and without a significant impact in terms of overall survival [26-29]. In this setting, the use of PLD is supported only by subgroup analysis of different clinical trials, where even in the absence of a significant superiority in term of survival a better toxicity profile was found [26-29]. A phase III trial, specifically designed for platinum-refractory setting, did not show any advantage for PLD in term of overall survival (OS) and toxicity when compared to gemcitabine [28].

The purpose of our retrospective analysis is to assess the actual impact of PLD in the management of OC patients in our Institution.

## Patients and methods

We performed a retrospective analysis on 108 patients with confirmed histology of serous papillary high grade OC, followed at our Institution between 2001 and 2011. Clinical data have been retrieved from medical records and entered into an electronic database. Eligibility criteria included histologically-confirmed diagnosis of serous papillary adenocarcinoma, age >18 years, ECOG performance status 0–1, adequate renal and liver function and no major comorbidities. 80/108 (74%) patients were in stage III/IV and 55 of them (51%) received a second-line treatment. Different OC prognostic factors were evaluated in our series: age, performance status, comorbidities, previous malignancies, date of first diagnosis, stage, grade, and presence/absence of metastases at diagnosis, site of metastases, CA125 levels (baseline, during chemotherapy, at eventual disease relapse), first-line chemotherapy with response and toxicity, eventual II look surgery, any subsequent line of chemotherapy. Response to chemotherapy has been reported according to Response Evaluation Criteria In Solid Tumors (RECIST). Patients were monitored every 3 months. When disease progression was thought for increasing CA125 or for the onset of clinical symptoms, all patients underwent total body computed tomography scan (TC scan) and/or other imaging procedures to assess the status of disease. For PLD efficacy analysis, patients were divided into 2 subgroups: *i*) 30 patients treated with PLD for recurrent or progressive disease, and *ii*) 25 patients never treated with PLD, which were evaluated for survival and treatment endpoints. In the decision-making, PLD treatment was selected on the basis of platinum-sensitivity and toxicities reported to prior chemotherapy. PLD was administered at the dose of 40–50 mg/mq q28 days for a median of 6–8 cycles, according to the international guidelines. The use of granulocyte colony stimulating factors and erythrocyte stimulating factors was at physician choice. Both OC patient groups underwent a mean of 3 treatment lines with a median follow-up of 60 months. The study was performed according to the bioethics standard of our Institution and all patients at first admission visit had provided consent to anonymous data management for clinical research.

### Statistical analysis

In order to avoid selection bias, data were collected by two independent investigators (N.S. and D.C.) and the missing information have been subsequently discussed and solved by the aid of an arbiter (P.T.). Primary endpoint was OS in all patients and specifically in patients treated with PLD in all treatment lines (second-line and/or subsequent lines) as compared to no-PLD treatment. Other endpoints were PFS, response rate (RR) and toxicity. OS was defined as the time elapsed between the start of treatment and the date of death. PFS is intended as the time from start of treatment to progression or death. The time to relapse of ovarian cancer after platinum treatment was assessed by CA125 and imaging (CA125- or image confirmed-PFS which undergone separate analysis) while responses were evaluated by RECIST criteria. The differences between the patients’ baseline characteristics were analyzed with the Wilcoxon test and the method of Chi-square. Differences were considered statistically significant with p value ≤0.05. Survival analysis and correlations between patients’ baseline characteristics and toxicity were evaluated by Kaplan-Meier curves and Log-Rank test statistics in the univariate analysis. Subsequently variables with significant p value were entered into a multivariate analysis model according to Cox proportional hazards model to analyze the role of confounding factors [30]. The relative hazard ratios (HR) with 95 percent confidence intervals (95% CIs) were calculated using SPSS (version 19) statistical package.

## Results

In Table 1, the baseline characteristics of 108 patients enrolled in our retrospective analysis are reported. All patients were treated at our University Cancer Center from January 2001 to December 2011. Median age was 59 years, median ECOG PS= 0. 80% of patients presented in advanced stage with lymph node involvement (FIGO IIIC) and 20% of them had grade 2 histology and 70% grade 3. Median OS in patients in early stage of disease was 80 months, with a more favorable outcome as compared to literature data. Age, performance status and stage at diagnosis were comparable to inclusion criteria of major trials. About 60% of patients presented with comorbidities (eg, hypertension, diabetes mellitus type 2) grade 2 according to CTCAE criteria v 4.0. The median value of baseline CA125 (205 IU/ml) was calculated in the whole study population, and the patients were divided into 2 groups according to this cut-off. In a small subgroup of 32 patients it was possible to investigate the value of D-dimer as prognostic factor. 51% of the patient received a second-line treatment and about 25% underwent a third-line treatment. 20% of the patients underwent second-look surgery.

**Table 1:** Main patient characteristics

**Characteristics**	**Number of patients (%)**
**Median age (59 years)**	
-Age between 18–59 years	55 (51)
-Age > 59 years	53 (49)
**Performance status (PS Ecog)**	
-0	80 (74)
-1	25 (23)
-2	3 (3)
**Stage at diagnosis (n)**	
-Stage I	22 (20)
-Stage II	6 (5)
-Stage III	49 (45)
-Stage IV	31 (29)
**Grading**	
-G1	11 (10)
-G2	26 (24)
-G3	71 (66)
**Median value of CA 125**	
CA 125 ≥ 205	46 (42)
Ca 125 < 205	43 (40)
**D-dimer**	
-Hig D-dimer value	17 (16)
-Low D-dimer value	15 (14)
**Comorbidity**	
-No comorbidity	31 (29)
-Grade 1 comorbity	10 (9)
-Grade 2 Comorbidity	67 62)
**Adjuvant treatment**	
-yes	17 (16)
-no	10 (9)
**First line treatment**	
-yes	64 (59)
-no	2 (2)
**Neoadjuvant treatment**	15 (14)

**Figure 1:**
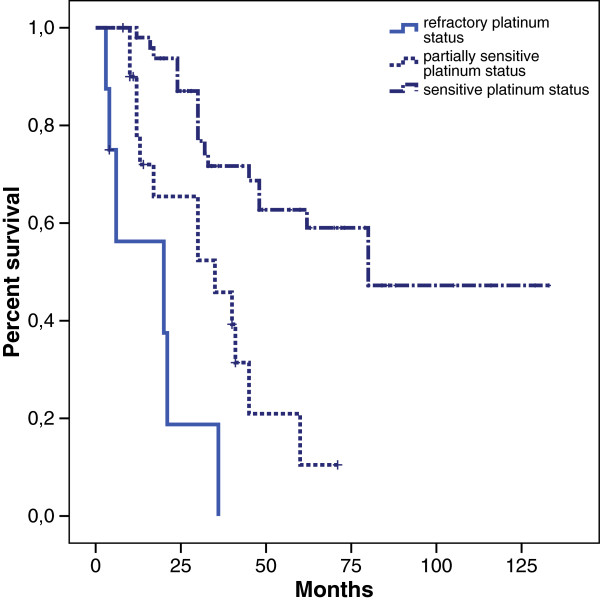
Kaplan Meier overall survival and response to platinum treatment.

**Figure 2:**
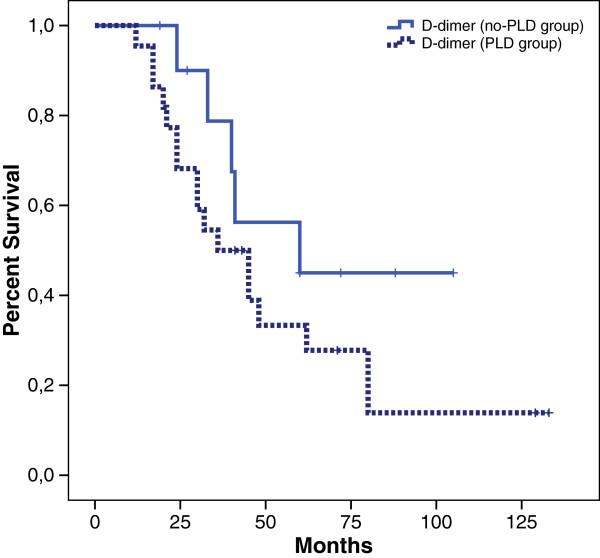
Kaplan Meier overall survival: D-dimer and PLD treatment.

Among 55 patients who received second-line chemotherapy, 23 patients presented platinum-resistant disease, 15 patients partially platinum-sensitive disease and 17 patients platinum-sensitive disease. Median OS of patients with recurrent disease was 48 months and median first line PFS was 12 months (Figure 1). Subsequently, we investigated the impact of each prognostic factor on survival. In particular, patients with the lower CA125 value at diagnosis had a better outcome in terms of PFS (17 *vs* 12 months) with an HR 0.40 (0.21 to 0.79, p = 0.008). This finding was also confirmed in OS (62 *vs* 35 months, HR 0.45 CI: 0.20 to 0.99, p = 0.049). OS evaluated according to platinum response was 30 months for refractory patients, 80 months for patients partially platinum-sensitive while median survival was still not reached for platinum-sensitive patients . A D-dimer value in normal ranges was associated (even if the statistical significance was not reached) with a longer PFS (28 *vs* 13 months; HR 0.35 CI: 0.06 to 1.77, p = 0.07) (Figure 2).

**Figure 3:**
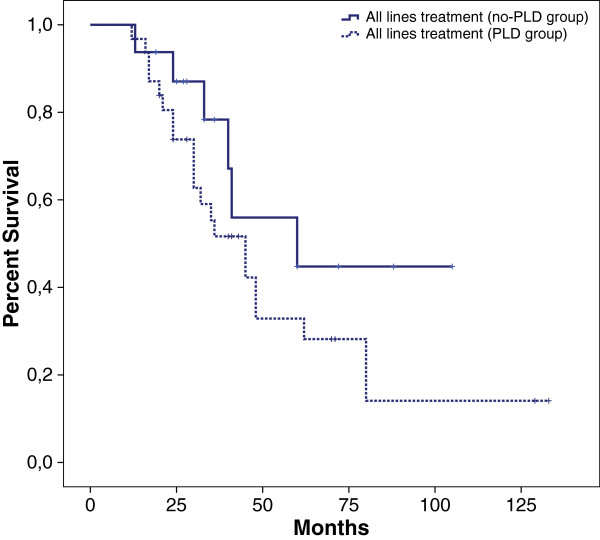
Kaplan Meier overall survival: PLD treatment in all lines treatment.

**Figure 4:**
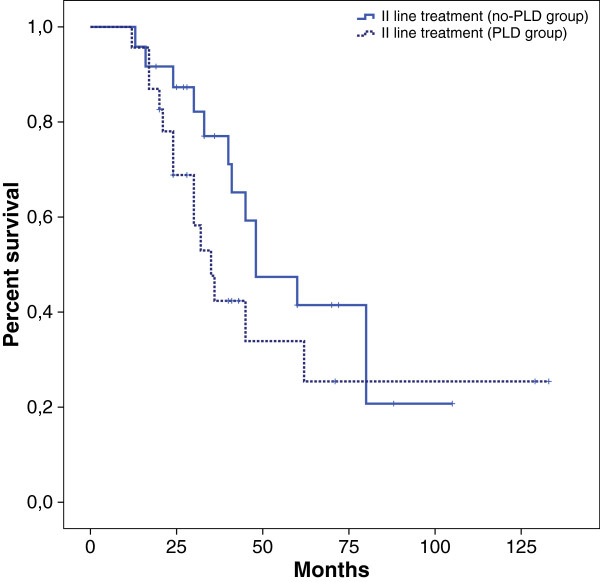
Kaplan Meier overall survival: PLD treatment in II line treatment.

We compared the outcome of patients treated with PLD along the course of their disease with control arm (patients distribution showed in Table 2) represented by patients never treated with this agent but treated with other drugs such as topotecan, gemcitabine, etoposide. Among them, 37% had a platinum-refractory status and 43% had partially platinum-sensitive status. Both groups shared similar baseline characteristics after the first line treatment (Table 3). PFS was 6 months in PLD group and 10 months in control arm (HR 0.57 CI: 0.30 to 1.06, p = 0.07). OS was 45 months *vs* 65 months respectively (HR 0.40 CI: 0.15 to 1.05, p =0.06) (Figure 3). We performed a further exploratory analysis to compare survival between patients treated with PLD in second line with patients treated with a different agent (control arm) (Figure 4). This analysis revealed an advantage in term of OS in the PLD-free series (35 *vs* 48 months). These results were not due to an asymmetric distribution of platinum-sensitive patients between PLD and no-PLD groups. Overall RR in II line treatment was 43% (13% in PLD group *vs* 57% in PLD-free group).

**Table 2:** PLD use beyond first line treatment

	**Patients (%)**
**II line treatment**	**55**
○ Pld	**22 (40%)**
○ other therapy	**33 (60%)**
**Among all treatment lines**	
○ Pld	**30 (54%)**
○ other therapy	**25 (46%)**

**Table 3:** Treatment choice on the basis of platinum sensitivity status (all lines treatment)

	**PLD (30 patients)**	**Other therapy (No PLD) 25 patients**
**Platinum sensitive**	9 (30 %)	8 (32%)
**Partially-platinum sensitive**	10 (33%)	5 (20%)
**Platinum refractory**	11 (37 %)	12 (48%)

### Toxicity

Table 4 reports toxicities recorded for PLD arm compared to other treatment arm in our study. About 27% of patients treated with PLD experienced grade 2 or 3 toxicities. The most common toxicities were neutropenia (14%), thrombocytopenia (7%), anemia (1%), hand–foot syndrome (5%), mucositis (5%). In control arm, toxicities were comparable with literature data on the basis of selected treatment.

**Table 4:** Toxicity in subsequent lines of treatment (Grade ≥ 2)

	**PLD arm**	**Other treatment**
Neutropenia	14%	20%
Thrombocytopenia	7%	12%
Anemia	1%	3%
Hand–foot syndrome	5%	0%
Oral toxicity	5%	3%
Neurological toxicity	1%	7%

## Discussion

The results of our retrospective study do no support the common belief that PLD is the first choice for recurrent or progressive OC treatment. Indeed, in our analysis, we were unable to demonstrate advantages in terms of PFS and OS for PLD administration at any time along the course of disease. However, platinum-sensitive patients took benefit from PLD more than the platinum-resistant group.

In our patient series, PLD given at any point indeed translated in a trend to worse outcome in term of both PFS and OS if compared to patients that never received this agent. While clinical trials indicated that PLD is better tolerated as compared to other drugs, no difference in toxicity was observed in the current study. In our series, 16 platinum-sensitive patients were treated according to CP schedule [20] and 6 patients received PLD. The latter group however, experienced a worse outcome. The common practice of PLD usage in the platinum-sensitive setting has been defined on subgroup analysis of major randomized trials. Indeed, some reports showed a 2 months advantage for PLD compared to topotecan in terms of PFS in platinum-sensitive subgroup only [26]. Subsequently, the non-inferiority phase III Calypso trial compared carboplatin-PLD (CD) *vs* carboplatin-paclitaxel (CP) in patients with platinum-sensitive recurrent OC. In this trial the primary end-point (PFS) was met and a superiority for CD regimen was suggested. However, this conclusion is not appropriate in the light of the non-inferiority trial design. Indeed, reported results are not sufficiently strong to support the PLD use as first choice in clinical practice. The cornerstone of platinum-sensitive OC treatment is currently represented by re-challenge with platinum which is clearly effective, with a response rate of about 50-60%. Meaningfully, the anti-VEGF monoclonal antibody bevacizumab is the only agent able to produce an improvement of 4 months in terms of PFS in combination to platinum-containing second line treatment [31]. In the platinum-resistant setting topotecan or gemcitabine may represent an adequate option despite a low response rate and a moderate bone marrow toxicity. Several trials compared PLD to gemcitabine in platinum-refractory patients and/or partially platinum-sensitive patients without showing advantage for PLD, but only a trend in terms of PFS for the subgroup of patients with a platinum-free interval (PFI) between 7 and 12 months [28].

In our experience, 18 patients with a partially platinum-sensitive status undergone a second line therapy. Of these, 13 patients were treated with a PLD-based schedule (80% of them in combination with trabectedine, TB) whereas 5 patients received other agents. Some authors demonstrated that TB in addition to PLD produces a 3 months increase in PFS [32]. However, in our series, the 5 patients in PLD-free group had a better outcome in terms of both PFS and OS (the small sample size may account for the lack of statistical significance).

Our study presents some limitations such as the small sample of patients, the heterogeneous therapeutic strategies, the long enrollment time, with the possible occurrence of selection bias. Moreover, our medical records do not include data about quality of life, an important factor in palliative setting. However, our findings are in agreement with a small retrospective study which included 43 patients treated with PLD and indicated only a marginal benefit in terms of survival for PLD in the platinum-resistant/refractory setting despite a considerable toxicity [33]. Furthermore it is important to underline that none of major trials reported a real advantage for PLD if compared to any other drug in terms of OS. Surprisingly, a recent phase III trial which compared PLD to patupilone showed a limited improvement in terms of OS for PLD in platinum-refractory OC [34]. These findings are difficult to interpretate taking into account the limited experience on patupilone. Interestingly, the AURELIA trial, designed for the platinum-refractory setting, investigated the effect of bevacizumab addition to single agent chemotherapy including PLD. In this trial the anti-VEGF monoclonal antibody produced almost a doubling (3.4 *vs* 6.7 months) in PFS but this advantage was indeed more relevant for weekly paclitaxel if compared to PLD and topotecan [35].

Our findings need to be considered in the general scenario of anthracycline-based treatment of OC. Recently, a report demonstrated an advantage in terms of PFS for PLD *vs* Olaparib in platinum-refractory patients with identified BRCA mutations, [36]. Moreover, others studies reported a correlation between functional homologous recombination deficiency (HRD) and clinical benefit from antracycline [37]. We did not find, however, any advantage from the administration of PLD in patients not selected for BRCA1 mutational status. During the last years, several trials debunked anthracycline role in the management of OC for a no clear advantage in efficacy at cost of an increased toxicity [38,39].

In particular, different trials evaluated the actual cost/effectiveness of chemotherapy beyond first line treatment. The effectiveness of chemotherapy is correlated to PFI: in platinum-sensitive patients PLD is cost/effective compared with paclitaxel or topotecan but less cost/effective if compared with carboplatin/paclitaxel combination; in platinum-refractory patients best supportive care could be cost/effective compared to PLD, topotecan or gemcitabine combination in unfit patients [40,41].

In conclusion, our retrospective experience in a consecutive series of serous papillary OC challenges the current believes on PLD activity in OC. Further prospective studies are needed to confirm our results, and molecular analysis as well as investigations on the peritoneal microenvironment are eagerly awaited to shed light on the molecular basis of the response to PLD, in order to identify patients who would gain benefit from the treatment with this agent.

## Competing interests

The autthors declare that we have no competing interests.

## Authors’ contributions

NS, PFT and PT designed the study, NS, AS, LF and SG provided and analyzed clinical data, NS, DC and CB performed statistical analysis. NS, PT and PT wrote the manuscript. All authors read and approved the final manuscript.
